# Decay Rates of Plasmonic Elliptical Nanostructures via Effective Medium Theory

**DOI:** 10.3390/nano11081928

**Published:** 2021-07-27

**Authors:** Mohammed Gamal, Ishac Kandas, Hussein Badran, Ali Hajjiah, Mufasila Muhammed, Nader Shehata

**Affiliations:** 1 Center of Smart Nanotechnology and Photonics, SmartCI Research Center, Alexandria University, Alexandria 21544, Egypt; eng-mohammed.gamal.1520@alexu.edu.eg (M.G.); ishac@vt.edu (I.K.); 2 Department of Engineering Mathematics and Physics, Faculty of Engineering, Alexandria University, Alexandria 21544, Egypt; 3 Department of Electrical Engineering, College of Engineering and Petroleum, Kuwait University, Safat 13133, Kuwait; badran.hussein23@gmail.com (H.B.); Dr.Hajjiah@gmail.com (A.H.); 4 Kuwait College of Science and Technology, Doha Area, 7th Ring Road, Safat 13133, Kuwait; mufasila@gmail.com; 5 USTAR Bioinnovation Center, Utah State University, Logan, UT 84341, USA; 6 The Bradley Department of Electrical and Computer Engineering, Virginia Tech, Blacksburg, VA 24061, USA

**Keywords:** decay rate, plasmonic nanostructures, effective medium theory, ellipsoids, moth-eye

## Abstract

This paper investigates the spontaneous decay rate of elliptical plasmonic nanostructures. The refractive index was analyzed using the effective medium theory (EMT). Then, the polarizability, spontaneous radiative, non-radiative decay rate, and electric field enhancement factor were characterized for the targeted elliptical nanostructures at different aspect ratios. All of the optical analyses were analyzed at different distances between the excited fluorescent coupled atom and the plasmonic nanostructure (down to 100 nm). This work is promising in selecting the optimum elliptical nanostructure according to the required decay rates for optical conversion efficiency control in energy harvesting for solar cells and optical sensing applications.

## 1. Introduction

Nanotechnology has been a major focus of scientific attention over past decades, exhibiting an exponential growth for the number of publications in plasmonic nanostructures (NSs) and light scattering in several such plasmonic materials like silver nanorods and gold nanoparticles [1]. Plasmonic nanoparticles (NPs) exhibit many useful properties, especially metallic NPs, due to the surface plasmon resonance (SPR) effect [2,3]. This SPR phenomenon causes the resonance of collective electrons, known as surface plasmons, when polarized light hits a metallic film at the interface of media with different refractive indices which allows for a major increase in the light absorption and light scattering to the other wavelengths that does not matched with the resonance of the plasmonic material. This remarkable optical property makes plasmonic NPs a major subject of interest in the study of optics and different applications in electronics, communications, and biomedicine [4].

Recently, the light scattering has become a very essential tool for sensing application in several fields such as the material and the biomedical science [5]. However, the scattering properties of the plasmonic nanoparticles are very good, the gold and silver metal nanoparticles widely used in scattering applications [6]. One advantage of these material that they are non-toxic, but it is relatively expensive [5,7]. Thus, the plasmonic nanoparticles are used in Surface enhanced Reman Spectroscopy (SERS), which is a powerful method for detecting tumors and cancers cells in alive body [5,8,9,10,11,12]. For the material science applications, the light scattering plays a significant rule to measure the morphology and the characterizations of the materials [13]. There are many devices uses this technology such as the Light Scattering Spectroscopy (LSS) to measure the spectral power density of the material surface [14], the Phase Analysis Light Scattering (PALS) measures the motilities of the colloidal particles [13,15], the Laser Doppler Anemometry (LDA) to investigate the dynamic fluid [16], the Forced Rayleigh Scattering (FRS) to measure the diffusion of the materials [17,18].

The need to compute the effective properties for the plasmonic nanoparticles has led to establish a theory that can calculate the micro and macro optical properties for arbitrary nanoparticle shape in a homogenous medium. So that the scientists forge many theoretical methods to do that, whereas the most important one is the Effective medium theory (EMT) [19]. In 1904, Maxwell–Garnett provided the EMT, whereas it makes a homogeneity between the impeded nanoparticles and the certain medium. In addition, the EMT makes a very good approximation to the complex electromagnetic medium. Thus, the EMT has also succeeded to calculate the permittivity for this effective medium by taking the volume fractions f into account for every individual particle in this theoretical medium [20,21].

In 1916, Einstein provided the equilibrium emission theory for an excited quantum state has emitted a spontaneous and stimulated light to calculate the spontaneous emission decay rate [22]. Then, Dirac had predicted the theory of emission for quantum mechanics according to his probability of the photon emission theory [23]. As Purcell discovered that the resonant cavity affects the atomic fluorescence by controlling the spontaneous decay rate. Recently, we know that the material geometry shape also can affect the influence of the spontaneous decay rate. Thus, we can control the spontaneous emission by controlling shape of the nanoparticle. This principle is widely used to enhance light sources [19]. For decay rates of fluorescence emission, the SPR resonate cavity can effectively enhance, either linearly or non-linearly, the atomic fluorescence decay rate depending on the geometry of the metallic nanostructures [24,25]. Therefore, the investigation of the interaction of single atoms, molecules, and quantum dots with optical fields in the presence of a nanobody becomes essential to improve the optical efficiency of fluorescence emission [26]. Besides, the spontaneous emission of the single molecule could be used as a normalized light and utilized to study the sized nano-bodies [26,27]. Consequently, the spontaneous decay rate is helpful to determine the measured quantity of nano-bodies at fluorescent detection along with the identification of a single molecule by using the scanning microscopes [28]. Moreover, the metallic nano-radiator quantum dot can be employed as an amplifier to the plasmonic surface by using the emission radiation [29,30]. The plasmonic metallic nanostructures; such as gold, silver, and copper can be embedded in many applications such as solar cells [31,32,33,34,35,36], up conversion [32,37,38,39], light emitting diodes (LEDs) [40,41], lasers and laser printing [42,43,44,45], sensors and photodetectors [46,47,48,49]. Moreover, the plasmonic nanoparticles aid to slow down the speed of light which can enhance both absorption efficiency and optical coupling in the waveguide-cavity for optical communication networks and sensing applications [25,48,49,50,51,52,53]. In addition, the spontaneous emission is wildly used to control and tuning the light sources and its efficiency [30,54]. Also, the emission of the nanobody that is emitted from the single molecule can be applied to influence the DNA structure without using any addition to the fluorescent markers [55]. Thus, it is important to know how to match the fluorescent properties of the NPs with the detected molecule and consequently to improve the selectivity of the detection [26].Different plasmonic nanostructures, such as gold and silver formed as nanorod, spheroid, sphere, ellipsoid, and half ellipsoid (moth-eye) lead to enhance the electric field around them to the surrounding medium which improves the optical coupling of any fluorescent emission matched with surface plasmonic resonance (SPR) wavelength [56,57,58,59,60].

In this paper, we used the EMT to calculate the effective properties such as the effective RI and the effective polarizability in order to calculate both effective spontaneous radiative and non-radiative decay rates, and effective enhancement factor with taking the geometrical shape of the plasmonic nanostructures into account, such as ellipsoid, sphere, and spheroid. We analyzed this calculation at different distances between the excited atom and the plasmonic structure down to 100 nm, with detection of optimum ranges of plasmonic geometry’s aspect ratio, coupling wavelength, and distance.

## 2. Mathematical Background

### 2.1. Mathematical Interpertation of EMT

**Figure 1:**
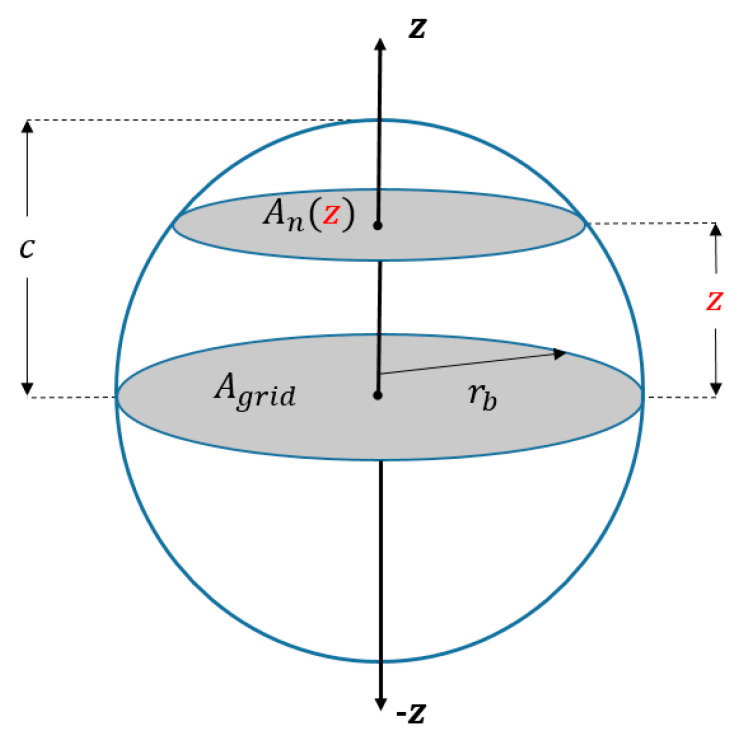
Shape geometry analysis for EMT calculations.

The EMT has been selected to model our selected NPs to obtain a generalized formula for the real part (n), and the imaginary part (k) of RI. This is performed by calculating an effective RI which is a function of the height or depth of the nanostructure, with focusing on NP geometry, as well as the refractive indices of both the NP and its surrounding medium. The effective RI for the ellipsoid and spheroid can be approximated by both of the following equations; Equations (1) and (2), respectively. This approximation is acceptable for small value of $L_{o}$ for the ellipsoid or large value of $L_{o}$ for the spheroid in order of close to zero and close to 1 for both ellipsoid and spheroid, respectively, as shown in details in Section S3 in Supplementary Materials [20,61]:

(1) $$n_{eff}\left(z\right)\approx n_{2}\cdot f\left(z\right)+n_{1}\cdot \left(1-f\left(z\right)\right)$$

(2) $${\left(n_{eff}\left(z\right)\right)}^{-1}\approx n_{2}{}^{-1}\cdot f\left(z\right)+n_{1}{}^{-1}\cdot \left(1-f\left(z\right)\right)$$

where both *n*_1_ and *n*_2_ are the RIs of the surrounding medium and NP, respectively, while *f*(*z*) is volume ratio which is function of the height with respect to the *z*-axis and could be expressed as $f\left(z\right)=A_{n}\left(z\right)/A_{grid}$, with *A_n_*(*z*) being the cross-sectional area of the NP at a specific height on the *z*-axis and *A_grid_* being the area of the NP arrangement circular grid as shown in Figure 1. Then, the effective RI can be written as Equation (3):

(3) $$n_{eff}\left(z\right)\approx n_{2}\cdot \frac{A_{n}\left(z\right)}{A_{grid}}+n_{1}\cdot \left(\frac{A_{grid}-A_{n}\left(z\right)}{A_{grid}}\right)$$

From Equation (3), both areas are expressed as $A_{grid}=\pi r_{b}^{2}$ and $A_{n}\left(z\right)=A_{grid}\cdot AHF\left(z\right),$ where rb is the base radius of the grid and *AHF*(*z*) represents the area height factor, ranging from 0 to 1. Additionally, since the base area of all NPs is also circular, the equation can be further simplified to obtain the following expression [34]:

(4) $$n_{eff}\left(z\right)\approx AHF\left(z\right)\cdot (n_{2}-n_{1})+n_{1}$$

The parameter of *AHF*(*z*) is a function of the geometrical curvature of the NPs as a function of the height along the *z*-axis. Since all our shape geometries follow the equation:$\frac{x^{2}}{a^{2}}+\frac{y^{2}}{b^{2}}+\frac{z^{2}}{c^{2}}=1$, as clarified in Figure 1, the *AHF* was derived to be $AHF\left(z\right)=1-{\left(\frac{z}{c}\right)}^{2}$ where *c* is constant. Thus, the effective RI for an ellipsoid on a circular grid can be approximated by Equation (5), which is valid for all ellipsoids, including spheres. In our case, this equation will be applied on our ellipsoid, sphere, and spheroid nanoparticles.

(5) $$n_{eff,ellipsoid}\left(z\right)\approx \left(1-{\left(\frac{z}{c}\right)}^{2}\right)\cdot (n_{2}-n_{1})+n_{1},-c\leq z\leq c$$

Utilizing Equation (5), an effective RI sweep can be performed and calculated at any depth, ranging from the RI of surrounding medium value *n*_1_ at *z* = *c*, to the RI of NP value *n*_2_ at *z* = 0 and back to the RI of surrounding medium value *n*_1_ at *z* = *c*.

According to the Electromagnetic theory, the RI of a material is a dimensionless complex number, which describes how the light being fast or bending when it goes throw the material based on EMT. We calculated the value of RI $\left(n_{t}\right)$ based on the real RI (n) and the extinction coefficient (k) according to the following equation $n_{t}=n\;-\;ik$, whereas the real RI is related to the velocity of light waves, while the extinction coefficient is related to the absorption or the damping of an oscillator. Then, we can introduce the permittivity in both Equations (6) and (7).

(6) $$\varepsilon \left(\omega \right)=n_{t}{}^{2}$$

(7) $$\varepsilon \left(\omega \right)=\left(n^{2}-k^{2}\right)+i2\;\left(nk\right)$$

where ε(ω) is the permittivity of NP which depends on frequency. Therefore, the polarizability $\alpha_{z}\left(\omega \right)$ can be calculated from the following equation; Equation (8) [21,62]:

(8) $$\alpha_{z}\left(\omega \right)=\frac{abc}{3}\frac{\left(\varepsilon \left(\omega \right)-\varepsilon_{m}\right)}{\varepsilon_{m}+L_{o}\left(\varepsilon \left(\omega \right)-\varepsilon_{m}\right)}$$

where $\alpha_{z}\left(\omega \right)$ is the polarizability. The parameters a,b, and c are the dimensions of the NP in the x, y, and z directions, respectively, $\varepsilon_{m}$ is the surrounding medium permittivity, which is assumed to be 1.5, and $L_{o}$ is the depolarization factor. In the case of both ellipsoid and spheroid, we considered that a=b and the depolarization factor is expressed according to the Equations (9)–(11) listed in Table 1.

Where the mathematical factor $\xi_{o}$ is kept as the positive root of $\xi_{o}{}^{2}$. It is a function of the atom position coordinates with assuming that the coupled atom is directly on the surface of the plasmonic surface. This factor differs from one shape to another and its value is calculated from the equations mentioned in the Table 1 [26,62]. Now, to calculate the general root ξ for the atom located outside the NP, we consider the following cubic equation in Cartesian coordinates (*x*, *y*, *z*):

(12) $$\frac{x^{2}}{a^{2}+\xi }+\frac{y^{2}}{b^{2}+\xi }+\frac{z^{2}}{c^{2}+\xi }=1$$

where $\xi >\xi_{o}$, if the atom is located outside the surface of nanoparticle, and ξ=0 if it is located inside the nanoparticle, which means that the decay rate is independent of the position of the atom inside nanoparticle [26,62]. At any arbitrary value of x, there is a possibility to have plasmonic resonances for a series of different shaped ellipsoids. Therefore, a small change of the ellipsoid shape is expected to result in a change in the spontaneous emission decay rate.

### 2.2. Radiative Decay Rate

Moving to the total decay rate ($\gamma_{tot})$, which is the sum of both radiative decay rate $\gamma_{rad}$ and radiation-less decay rate $\gamma_{nonrad}$. $\gamma_{rad}$ is related to the energy of free photons that emitted, whereas $\gamma_{nonrad}$ is the non-radiative decay rate that describes the losses inside the NP, related to the imaginary part of dielectric constant [54]. This section may be divided by subheadings. It should provide a concise and precise description of the experimental results, their interpretation, as well as the experimental conclusions that can be drawn.

(13) $$\gamma_{tot}=\gamma_{rad}+\gamma_{nonrad}$$

For mathematical interpretation of decay rates, the momentum dipole between the plasmonic NP and the fluorescent atom, which is affected by the plasmonic field, is assumed to be located and oriented along *z*-axis and then the normalized decay rate equation is simplified as presented in Equation (14):

(14) $${\left(\frac{\gamma }{\gamma_{o}}\right)}_{z}{}^{rad}={\left|1+\frac{1}{2}abc\;\left(1-\varepsilon_{zz}\right)\left(\frac{\varepsilon -1}{\varepsilon -\varepsilon_{zz}}\right)\;\times \left(\frac{2}{z\sqrt{\left(z^{2}+a^{2}-c^{2}\right)\left(z^{2}+b^{2}-c^{2}\right)}}-I_{c}\left(\xi \right)\right)\right|}^{2}$$

where ε is the permittivity of NPs. The integral $I_{c}\left(\xi \right)$ and $\varepsilon_{zz}$ are defined by the following relations:

(15) $$I_{c}\left(u\right)=\overset{\infty }{\underset{\xi }{\int }}\frac{du}{\left(c^{2}+u\right)\sqrt{\left(a^{2}+u\right)\left(b^{2}+u\right)\left(c^{2}+u\right)}}$$

(16) $$\varepsilon_{zz}=1-{\left(\frac{1}{2}\;\;abc\;I_{c}\right)}^{-1}$$

**Figure 2:**
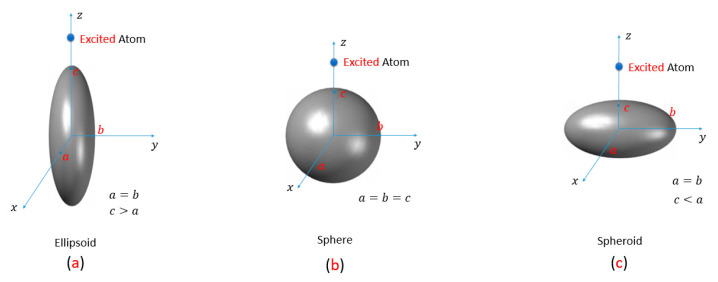
Simple sketch of the geometry of: (**a**) ellipsoid, (**b**) sphere, and (**c**) spheroid. The excited atom is located at different distances (**d**) from the close surface of the NP.

Then, the analysis procedure is to solve the integration of $I_{c}\left(u\right)$ for both ellipsoid and spheroid shapes then apply it in Equation (14), to obtain the normalized radiative decay rate for the structures of ellipsoid, sphere, and spheroid. More details about the mathematical solution of this integral is presented in Section S1 (Supplementary Materials). The coupled excited atom is considered to be located at different distances away from the surface of each geometry shape. In the case of the ellipsoid, where $L_{o}\to 0$ is approached to be a cylinder and the excited atom is located along its major axis. On the other hand, $L_{o}\to 1$ is approached to be a disc in the case of spheroid and the exited atom is located along its minor axis, as shown in Figure 2. To calculate the normalized decay rate for the ellipsoid and the spheroid, we substitute with c>a for the ellipsoid to determine the value of the $I_{c}\left(\xi \right)$ integration as shown in Equation (15); then, similarly by substitution when c<a for the spheroid. For both ellipsoid and spheroid geometrical cases, the integral $I_{c}$ is expressed as concluded in both Equations (17) and (18), respectively.

(17) $$I_{c}\left(\xi \right)=\frac{2}{c^{2}-a^{2}}\left[\frac{1}{2}\;\;ln\left(\frac{\sqrt{\frac{\xi +c^{2}}{c^{2}-a^{2}}}-1}{\sqrt{\frac{\xi +c^{2}}{c^{2}-a^{2}}}+1}\right)+\frac{1}{\sqrt{\xi +c^{2}}}\right]$$

(18) $$I_{c}\left(\xi \right)=\;\frac{2}{c^{2}-a^{2}}\left[\frac{1}{\sqrt{a^{2}-c^{2}}}\left(\frac{\pi }{2}-\tan^{-1}\left(\sqrt{\frac{\xi +c^{2}}{a^{2}+c^{2}}}\;\;\right)\right)+\frac{1}{\sqrt{\xi +c^{2}}}\right]$$

To determine the value of the factor ξ we apply Equation (12) to the location of the excited atom at *z*-axis, then, we get that $\xi =z^{2}-c^{2}$. Then, we substitute in the previous Equations (17) and (18) to obtain the radiative decay rate in Equation (19), as proved inside the Supplementary Materials, Section S2:

(19) $${\left(\frac{\gamma }{\gamma_{o}}\right)}_{z}{}^{rad}={\left|1+\frac{1}{2}\;abc\;\left(1-\varepsilon_{zz}\right)\left(\frac{\varepsilon -1}{\varepsilon -\varepsilon_{zz}}\right)\;\times \left(\frac{2}{z\sqrt{\left(z^{2}+a^{2}-c^{2}\right)\left(z^{2}+b^{2}-c^{2}\right)}}-I_{c}\left(z^{2}-c^{2}\right)\right)\right|}^{2}$$

whereas the $I_{c}\left(z^{2}-c^{2}\right)$ integrals for the ellipsoid and the spheroid are presented in both Equations (20) and (21), respectively. However, the same Equation (15) could not be applied for the sphere because the value of the integration $I_{c}\left(\xi \right)$ tends to infinity, so that another approximation is essential in the sphere case as shown in Equation (22) [26].

(20) $$I_{c}\left(z^{2}-c^{2}\right)=\;\frac{1}{c^{2}-a^{2}}\left[\frac{1}{\sqrt{c^{2}-a^{2}}}\frac{1}{2}\;\;ln\left(\frac{\sqrt{\frac{z^{2}}{c^{2}-a^{2}}}+1}{\sqrt{\frac{z^{2}}{c^{2}-a^{2}}}-1}\right)+\frac{2}{z}\right]$$

(21) $$I_{c}\left(z^{2}-c^{2}\right)=\frac{2}{c^{2}-a^{2}}\left[\frac{1}{\sqrt{a^{2}-c^{2}}}\left(\frac{\pi }{2}-\tan^{-1}\left(\sqrt{\frac{z^{2}}{a^{2}+c^{2}}}\;\;\right)\right)+\frac{1}{z}\right]$$

(22) $${\left(\frac{\gamma }{\gamma_{o}}\right)}_{r}{}^{rad}={\left|1+2\;\frac{\varepsilon \left(\omega \right)-1}{\varepsilon \left(\omega \right)+2}{\left(\frac{a}{r}\right)}^{3}\right|}^{2}\;\approx \;{\left|\frac{3\;\varepsilon \left(\omega \right)}{\varepsilon \left(\omega \right)+2}\right|}^{2}\;\;\;,\;\;\;r\approx a.$$

### 2.3. Non-Radiative Decay Rate

In this section, the non-radiative decay rates are presented for the different geometry shapes including ellipsoid, sphere, and spheroid. In contrast tothe radiative decay rate, and the non-radiative decay rate depend on the nanoparticle plasmonic mode and the distance between the excited atom and the surface of the nanoparticle. We consider that the distance between the excited atom and the nanoparticle is constant. Then, the first three modes with n=1, 2, and 3 are taken to use the mentioned expressions of Equation (23) for both the ellipsoid and the spheroid [62] and Equation (24) for the sphere [26], to calculate the normal non-radiative decay rate.

(23) $${\left(\frac{\gamma }{\gamma_{o}}\right)}_{normal}^{nonrad}=\frac{3}{8\;{\left(k_{o}\Delta \right)}^{3}}\;im\frac{\varepsilon \left(\omega \right)-1}{\varepsilon \left(\omega \right)+1}{\left(\frac{\gamma }{\gamma_{o}}\right)}_{tang}^{nonrad}=\frac{1}{2}{\left(\frac{\gamma }{\gamma_{o}}\right)}_{normal}^{nonrad}$$

(24) $${\left(\frac{\gamma }{\gamma_{o}}\right)}_{r}{}^{nonrad}=\frac{3}{8\;{\left(k_{o}\Delta \right)}^{3}}\;im\frac{\varepsilon \left(\omega \right)-1}{\varepsilon \left(\omega \right)+2}$$

where Δ is the distance between the exited atom and the NP and $k_{o}$ is the wave number which is defined as $\sqrt{\varepsilon_{m}}\frac{\omega }{c}$ [9,37], where ω is the angular frequency and c is the speed of light.

### 2.4. Electric Field Enhancement Factor

The local electric field is enhanced as a result of more optical polarization corresponding to coupling with plasmonic resonance [62]. In linear photoluminescence, the electric field enhancement (*γ_E_*) is defined by Equation (25).

(25) $$\gamma_{E}={\left|\frac{E}{E_{o}}\right|}^{2N}$$

(26) $$\frac{E}{E_{o}}=\frac{\varepsilon \left(\omega \right)}{\left(1-L_{o}\right)\varepsilon_{m}+L_{o}\;\varepsilon \left(\omega \right)}$$

where *E* is the local maximum of electric field, and *E_o_* is the amplitude of the input source electric field and *N* is an exponent order which is approximated to be 2 [37] and Equation (26) is a general relation that is valid for all targeted geometries; ellipsoid, sphere, and spheroid. The polarization factor ($L_{o}$) varies from one shape to another as explained before in Table 1.

## 3. Results and Discussion

### 3.1. Refractive Index (n) and Extinction Coefficient (k)

**Figure 3:**
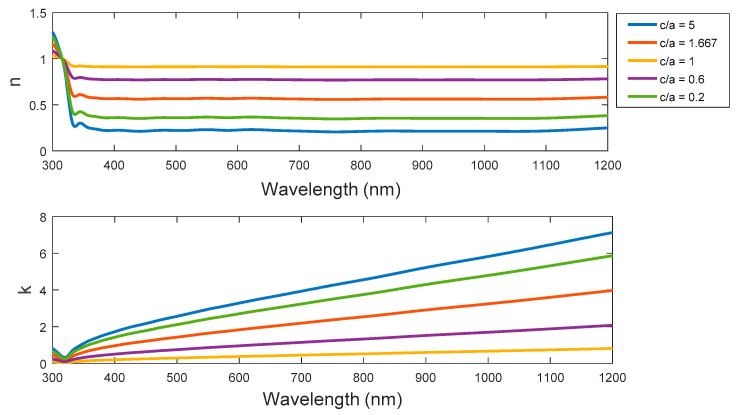
The real part of RI (n) and the extinction coefficient (k) for the ellipsoid, sphere, and spheroid at different aspect ratios.

Figure 3 shows both refractive index and extinction coefficient for ellipsoid, sphere, and spheroid at different aspect ratios. The saturation spectrum of the RI curves start at the wavelength of approximately 350 nm at all selected aspect ratios. The saturation level contrasts for the ellipsoid, sphere, and spheroid according to different aspect ratios with the reduced value of saturation levels for the ellipsoid at higher aspect ratios. Then, the RI value increases to the maximum at the case of sphere, and then decreases again according to the smaller aspect ratio of the spheroid. On the other hand, there is an incremental slope of the extinction coefficient k spectral curves at aspect ratios far from the sphere case of c/a = 1. In general, the slopes are higher in the case of ellipsoid than the slopes of the spheroid. As a result, the absorption and damping oscillation in case of ellipsoid is expected to be slightly larger than the spheroid.

### 3.2. Permittivity Analysis ε(ω)

**Figure 4:**
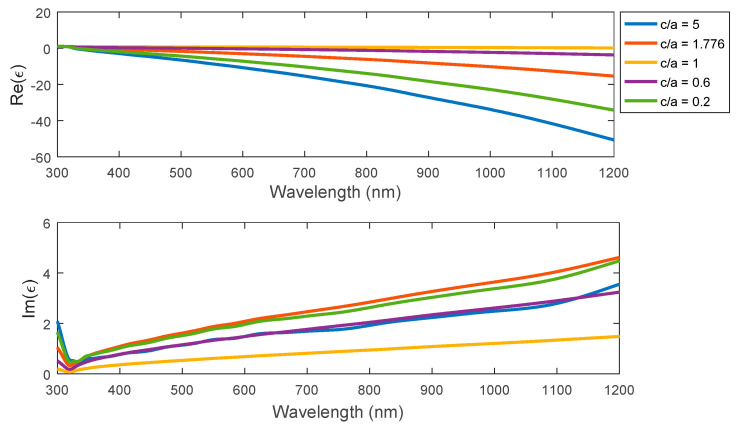
The permittivity for the ellipsoid, sphere, and spheroid in different (c/a) = 5, 1.667, 1, 0.6, 0.2.

Based on Equation (6), both the real and imaginary parts of the permittivity analysis have been presented as shown in Figure 4, with a similar behavior to the previously mentioned n and k spectral characteristics. In more detail, the spectral change of the real permittivity is larger with an increasing aspect ratio for the ellipsoid or a decreasing aspect ratio for the spheroid, with nearly constant permittivity for the case of sphere. For the imaginary part, the magnitude starts to be reduced at relatively shorter wavelengths in the near-UV region. However, for both visible and IR regions of the spectrum, the value of the imaginary part of permittivity has an increment behavior that is opposite to the real part performance.

### 3.3. Polarizabilty for the Studied Plasmonic Structures

**Figure 5:**
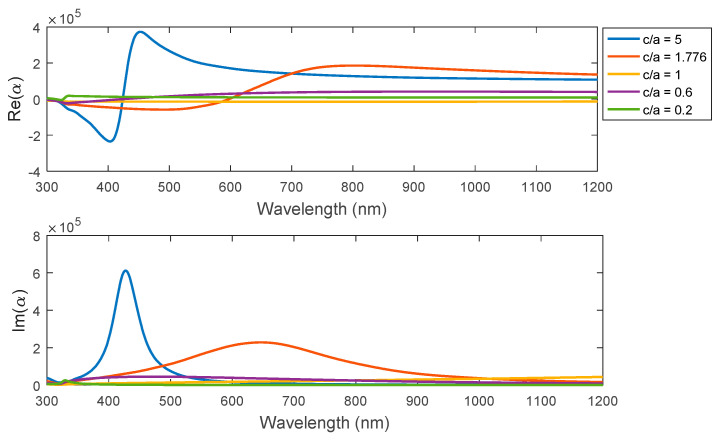
The real and imaginary parts of polarizability with different aspect ratios (c/a) for the studied elliptical geometries.

The polarizability curves of the real part, as shown in Figure 5. For the ellipsoid, the perturbation region is near the violet region, while the maximum perturbation is found at higher aspect ratios for the ellipsoid, according to larger directivity of the plasmonic geometry in the z-direction. In addition, the peak value of polarizability for the ellipsoid curves is decreasing while the aspect ratio decreases. In the same way, the maximum peak of the polarizability’s imaginary part becomes higher within a larger aspect ratio with a remarkable red-shift of the peak when reducing the aspect ratio of the ellipsoid part. On the other hand, there are no clear changes in polarizability’s peak value or the spectral shift for both sphere and spheroid geometries.

### 3.4. Radiative Decay Rate

**Figure 6:**
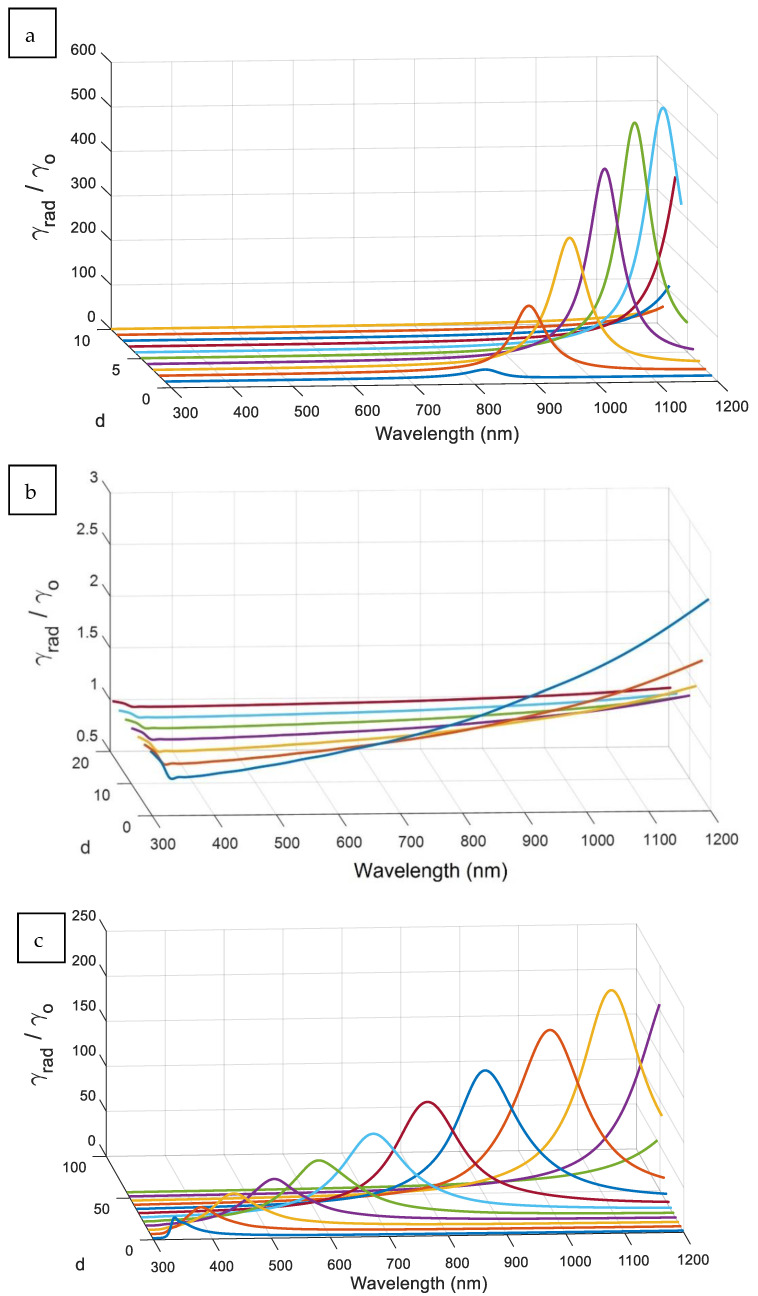
The radiative decay rate curves with the change of the distance d between the excited atom and the silver NPs with different aspect ratio (**a**) (c/a) = 5, (**b**) (c/a) = 1, and (**c**) (c/a) = 0.2.

The tuning peaks of the radiative decay rate, according to the change of the distance d between the excited atom and the Ag nanostructure, are shown in Figure 6 for different aspect ratios. At smaller distances (d) between the surface of the elliptical nanostructure and the excited atom, the radiative decay rate ($\gamma_{rad}$) peak value is decreased along with being spectrally-tuned to lower wavelengths for all aspect ratios. Generally, the spheroid peaks have a larger bandwidth compared to the ellipsoid peaks at different distances. The resonance peak intensity become greater and less broadening at both aspect ratios c/a = 5 and 0.2, whereas the structure is approaching to a nano-cylinder. For the ellipsoid with an aspect ratio of c/a = 5, the radiative decay rate covers the studied spectrum region from 300 to 1200 nm according to a smaller distance (d) up to 10 nm. In addition, it can be noticed that a smaller change in distance at higher aspect ratio of the ellipsoid nanostructure leads to a larger change in spectral peak tuning. In the case of spheroid, for example c/a = 0.2, the radiative decay rate covers the whole targeted spectrum with a larger needed distance with the excited coupled atom. Also, the change in distance is less effective in tuning of the spectral peak of the decay rate in the case of spheroid, compared to the ellipsoid. Therefore, the elliptical shape is more flexible in tuning the peak radiative decay rate, but given the control of the distance with the exited coupled atom to be relatively tighter.

**Figure 7:**
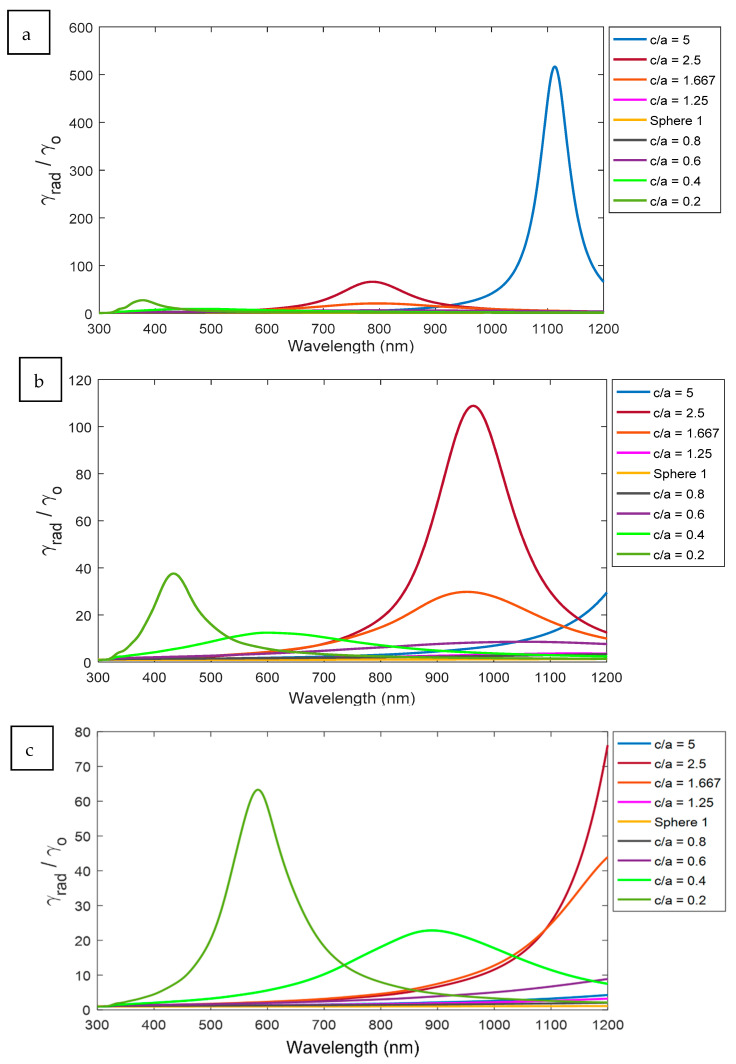
The radiative decay rate $\gamma_{rad}$ in a different aspect ratio c/a in different distances (**a**) d = 5, (**b**) d = 10, and (**c**) d = 20 nm.

In Figure 7, the spectrum of the radiative decay rate of the ellipsoid and the spheroid are presented at different aspect ratios for certain distances, as examples. The spectral peaks for ellipsoids are existed at much longer wavelength compared to spheroids. In addition, the highest intensities are found for higher aspect ratios of ellipsoid according to a better optical coupling probabilities between the sharper edge of the higher (c/a) ellipsoid and the excited atom.

**Figure 8:**
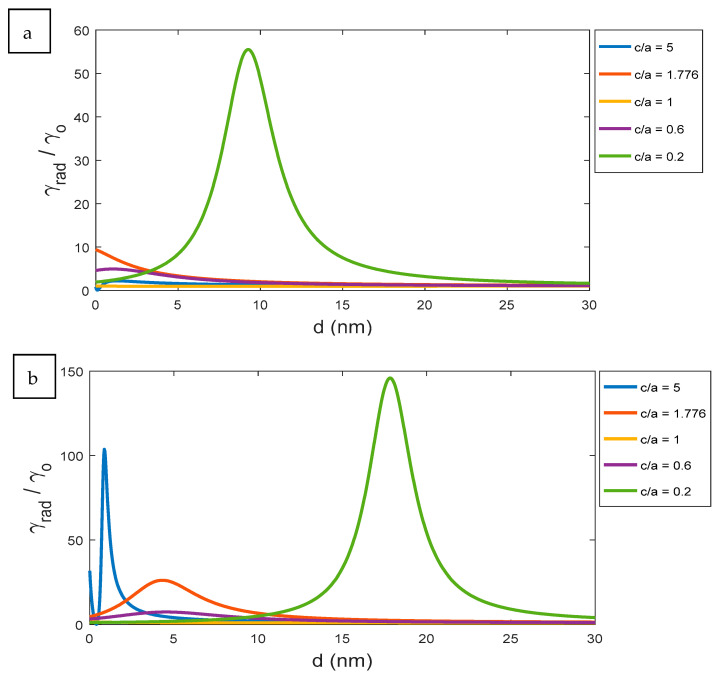
The radiative decay rate according to the change of the distance d for the ellipsoid, sphere, and spheroid at different wavelengths: (**a**) 550 nm and (**b**) 880 nm.

At certain wavelengths, the optimum design of the nanoparticle in different aspect ratios for visible and infrared wavelength can be determined, as shown in Figure 8. In the visible range closed to green emission at 550 nm, as an example, the spheroid of c/a = 0.2 becomes an optimum choice for the best radiative decay rate at a distance around 10 nm, as presented in Figure 8a. Within the IR region, such as 880 nm for example, both the ellipsoid and the spheroid offer optimum decay rates but at different designed distances. In more detail, the spheroid of aspect ratio 0.2 allows for a larger distance with the coupled excited atom up to 18 nm, as shown in Figure 8b. On the contrary, the ellipsoid offers a relatively large peak value of radiative decay rate, but at much smaller distances of less than 5 nm. Here, the flexibility in the selected distances and aspect ratios of the designed elliptical nanostructures give wide variety of applications such as energy harvesting and sensing within different spectral regions.

### 3.5. Non-Radiative Decay Rate Results

**Figure 9:**
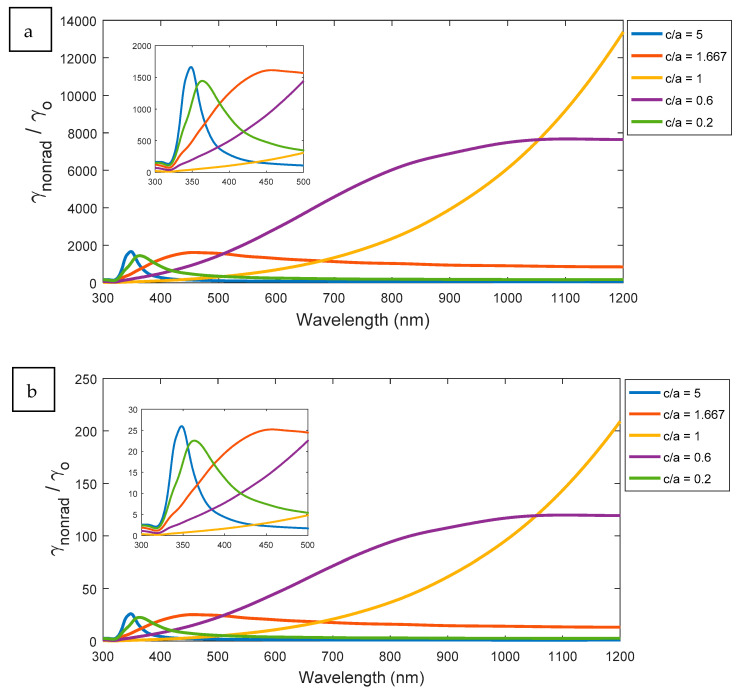
The Non-radiative decay rate for all aspect ratios at different dimension: (**a**) d = 5 and (**b**) d = 20 nm.

In this section, the non-radiative decay rates for the ellipsoid, sphere, and spheroid are discussed. The variation of non-radiative decay rate depends on the dimension ratio c/a. Both spheroid and ellipsoid with aspect ratios, such as 0.2 and 2.5, respectively, have maximum peaks at near UV regions, as shown in Figure 9. Unlike radiative decay rate analysis, the separation distance d does not affect the spectral behavior of non-radiative decay rate. Figure 9a,b shows that the spectral performance of non-radiative decay rates for different aspect ratios of elliptical nanostructures are similar at different distances to excited atoms, but with smaller values of the decay rates according to higher distance. This leads to reduce the losses effect or non-radiative coupling as long as the excited atom located away from the plasmonic elliptical nanoparticle.

**Figure 10:**
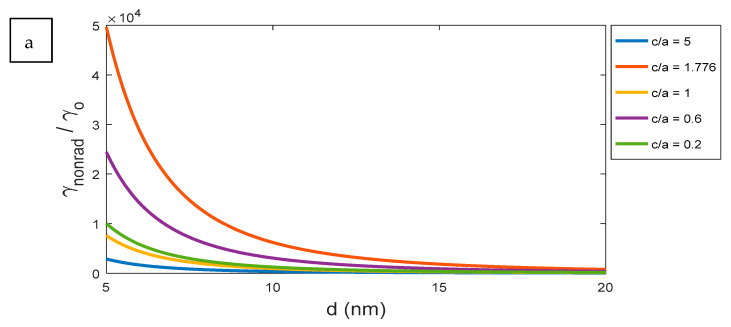

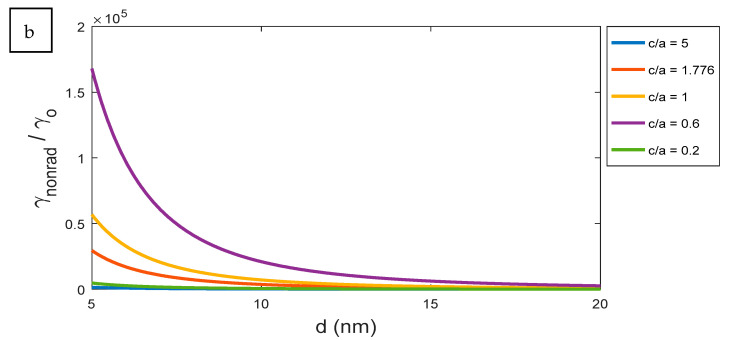
The effect on non-radiative decay rate $\gamma_{nonrad}$ with change the distance d of the excited atom from the surface at a certain wavelength: (**a**) 550 nm and (**b**) 800 nm.

The relation between non-radiation decay rate $\gamma_{nonrad}$ and distance d at certain wavelengths, such as 550 and 800 nm, are expressed in Figure 10. According to Equations (20) and (21), it shows that the distance *d* affects the non-decay rates by factor of $1/d^{3}$.

### 3.6. Electric Field Enhancement Factor

**Figure 11:**
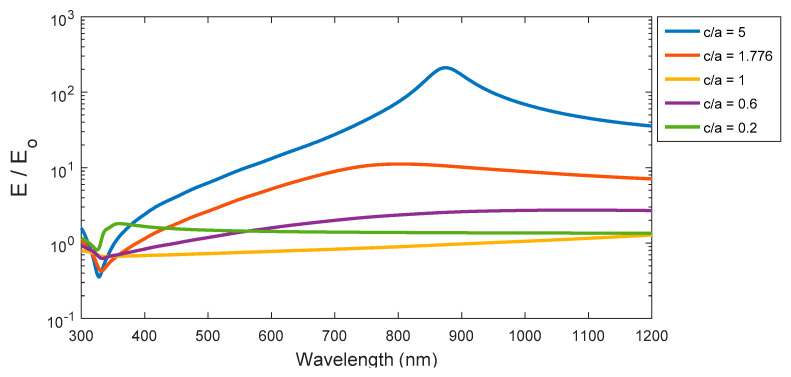
The enhancement factor (presented as logarithmic scale), for the ellipsoid, sphere, and the spheroid at different aspect ratios of elliptical structures.

The spectral curves of enhancement factor for ellipsoid, spheroid, and sphere are presented in Figure 11. The ellipsoid with c/a = 5 has the greatest peak value at wavelength 880 nm, then the peaks are decreased gradually with blue-shifted peaks toward the lowest wavelength whenever the aspect ratio decreases. The enhancement factor for both cases of sphere and spheroid are generally lower in intensity compared to the ellipsoid structure. Whereas, the enhancement factor of the sphere does not have a peak over the studied spectrum, but increases slightly with a longer wavelength. The enhancement of local electric field is increasing where the resonance occurs in prolate particles or near the spikes. That is clear in the cases of higher aspect ratios of the ellipsoid or smaller (c/a) for the spheroid.

## 4. Conclusions

Different plasmonic nanostructures including ellipsoid, sphere, and spheroid nanoparticles have been investigated to control the absorption and emission light using single molecule. Using the refractive index analysis via effective medium theory (EMT), different optical characterizations have been analyzed such as polarizability, spontaneous radiative/non-radiative decay rates, and enhancement factor. It has been found that we can control the maximum tuning of peak decay rates and enhancement factor according to different parameters including the aspect ratio of elliptical nanostructure and the distance between the excited atom and the plasmonic nanostructure surface. From our analysis, spheroids can be the optimum choice in near UV or visible spectrum with higher decay rates along with larger separation distances between elliptical nanostructures and excited atoms. However, the ellipsoid gives much larger radiative decay rate peaks in IR regions with smaller separation distances. This work is promising in selecting the optimum elliptical nanostructure according to the required decay rates for optical conversion efficiency control in energy harvesting and optical sensing applications.

## Figures and Tables

**Table 1:** Depolarization factor at different geometry conditions.

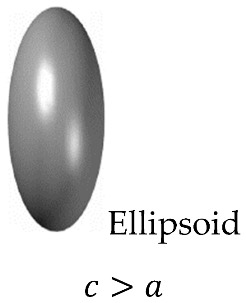	$L_{o}=\left(\xi_{o}{}^{2}-1\right)\left(\frac{\xi_{o}}{2}\ln\left(\frac{\xi_{o}+1}{\xi_{o}-1}\right)-1\right)$, $\xi_{o}{}^{2}=\frac{c^{2}}{c^{2}-a^{2}}$	(9)
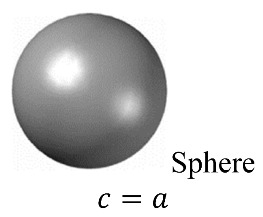	$L_{o}=\frac{1}{3}$	(10)
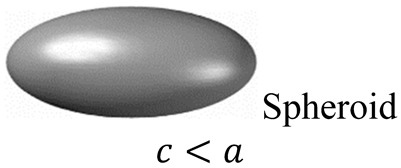	$L_{o}=\left(1+\xi_{o}{}^{2}\right)\left(1-\xi_{o}\tan^{-1}\left(\xi_{o}\right)\right)$, $\xi_{o}{}^{2}=\frac{c^{2}}{a^{2}-c^{2}}$	(11)

## Data Availability

Data is contained within the article or Supplementary Materials.

## Supplementary Materials

The following are available online at https://www.mdpi.com/article/10.3390/nano11081928/s1, Section S1. Proof of $I_{c}$ integral for both ellipsoid and spheroid, Section S2. Radiative decay rate for the ellipsoid and the spheroid using $I_{c}$ integral, Section S3. The general formula the effective medium theory.
