# Supplementary material for: Decay Rates of Plasmonic Elliptical Nanostructures via Effective Medium Theory
Source: Nanomaterials (Basel). 2021 Jul 27;11(8):1928. doi: 10.3390/nano11081928 (PMC8398447; doi:10.3390/nano11081928)
Supplement: Supplementary file 1 [file nanomaterials-11-01928-s001.zip › nanomaterials-1302579-SI.pdf]

nanomaterials

Supplementary Materials: Decay Rates of Plasmonic Elliptical Nanostructures via Effective Medium Theory

M. Gamal [1], I. Kandas [1,2], H. Badran [3], A. Hajjiah [3], M. Muhammed [4], and N. Shehata [1,2,4,5,6,*]

Section S1. Proof of I_c integral for both ellipsoid and spheroid

For the ellipsoid:

$$I_c = \int_{\xi}^{\infty} \frac{du}{(c^2+u)\sqrt{(a^2+u)(b^2+u)(c^2+u)}}$$

Note that: for the ellipsoid $a = b$

$$\therefore I_c = \int_{\xi}^{\infty} \frac{du}{(c^2+u)\sqrt{(a^2+u)^2(c^2+u)}} = \int_{\xi}^{\infty} \frac{du}{(u+c^2)^{\frac{3}{2}}|u+a^2|}$$

Because of a and b always are $+ve$.

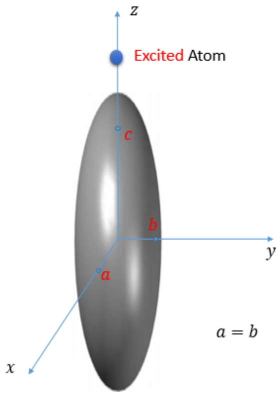

Figure S1. Atom located on z-axis at point z and having dipole momentum oriented along z-axis.

$$\therefore I_c = \int_{\xi}^{\infty} \frac{du}{(c^2+u)\sqrt{(a^2+u)^2(c^2+u)}} = \int_{\xi}^{\infty} \frac{du}{(u+c^2)^{\frac{3}{2}}(u+a^2)}$$

Let: $v = \sqrt{u+c^2} \rightarrow dv = \frac{1}{2\sqrt{u^2+c^2}} du \rightarrow du = 2\sqrt{u^2+c^2}\, dv$

$u = v^2 - c^2$ at $u = \xi \rightarrow v = \sqrt{\xi^2 + c^2}$

at $u = \infty \rightarrow v = \infty$

$$\therefore I_c = \int_{\sqrt{\xi+c^2}}^{\infty} \frac{2\sqrt{u^2+c^2}\, dv}{(v^2-c^2+c^2)\sqrt{u^2+c^2}(v^2-c^2+a^2)} = \int_{\sqrt{\xi+c^2}}^{\infty} \frac{2\, dv}{v^2(v^2-c^2+a^2)}$$

By partial fraction, we get:

$$\therefore I_{\xi} = 2 \int_{\sqrt{\xi + c^2}}^{\infty} \left(\frac{1}{(c^2 - a^2)(v^2 - c^2 + a^2)} - \frac{1}{(c^2 - a^2)v^2} \right) dv$$

$$= \frac{2}{(c^2 - a^2)} \int_{\sqrt{\xi + c^2}}^{\infty} \left(\frac{1}{(v^2 - c^2 + a^2)} - \frac{1}{v^2} \right) dv$$

By solving the 1st integral:

$$\int_{\sqrt{\xi + c^2}}^{\infty} \frac{1}{(v^2 - c^2 + a^2)} dv = \int_{\sqrt{\xi + c^2}}^{\infty} \frac{1}{v^2 - (c^2 - a^2)} dv$$

Let: $\omega = \frac{v}{\sqrt{c^2 - a^2}} \rightarrow d\omega = \frac{dv}{\sqrt{c^2 - a^2}} \rightarrow dv = \sqrt{c^2 - a^2}\, d\omega$

$v = \omega \sqrt{c^2 - a^2}$ at $v = \sqrt{\xi + c^2} \rightarrow \omega = \sqrt{\frac{\xi + c^2}{c^2 - a^2}}$

at $v = \infty \rightarrow \omega = \infty$

$$\therefore \int_{\sqrt{\xi + c^2}}^{\infty} \frac{1}{(v^2 - c^2 + a^2)} dv = \int_{\sqrt{\frac{\xi + c^2}{c^2 - a^2}}}^{\infty} \frac{\sqrt{c^2 - a^2}}{\omega^2 (c^2 - a^2) - (c^2 - a^2)} d\omega = \frac{1}{\sqrt{c^2 - a^2}} \int_{\sqrt{\frac{\xi + c^2}{c^2 - a^2}}}^{\infty} \frac{1}{\omega^2 - 1} d\omega$$

By using partial fraction, we get:

$$\therefore \int_{\sqrt{\xi + c^2}}^{\infty} \frac{1}{(v^2 - c^2 + a^2)} dv = \int_{\sqrt{\frac{\xi + c^2}{c^2 - a^2}}}^{\infty} \frac{1}{2} \frac{1}{\sqrt{c^2 - a^2}} \left(\frac{1}{\omega - 1} - \frac{1}{\omega + 1} \right) d\omega$$

$$= \frac{1}{2} \frac{1}{\sqrt{c^2 - a^2}} \left(\ln(\omega + 1) \Big|_{\sqrt{\frac{\xi + c^2}{c^2 - a^2}}}^{\infty} - \ln(\omega - 1) \Big|_{\sqrt{\frac{\xi + c^2}{c^2 - a^2}}}^{\infty} \right)$$

$$= \frac{1}{2}\frac{1}{\sqrt{c^2-a^2}}\left(ln(\infty) - ln\left(\sqrt{\frac{\xi+c^2}{c^2-a^2}}-1\right) - ln(\infty) + ln\left(\sqrt{\frac{\xi+c^2}{c^2-a^2}}+1\right)\right)$$

$$= \frac{1}{2}\frac{1}{\sqrt{c^2-a^2}}\, ln\left(\frac{\sqrt{\frac{\xi+c^2}{c^2-a^2}}+1}{\sqrt{\frac{\xi+c^2}{c^2-a^2}}-1}\right)$$

- By solving the 2nd integral:

$$\int_{\sqrt{\xi+c^2}}^{\infty} \frac{1}{v^2}\,dv = -\frac{1}{v}\Big|_{\sqrt{\xi+c^2}}^{\infty} = -\frac{1}{\infty} + \frac{1}{\sqrt{\xi+c^2}} = \frac{1}{\sqrt{\xi+c^2}}$$

∴ For ellipsoid

$$I_c(\xi) = \frac{1}{(c^2-a^2)}\left[\frac{1}{\sqrt{c^2-a^2}}\, ln\left(\frac{\sqrt{\frac{\xi+c^2}{c^2-a^2}}+1}{\sqrt{\frac{\xi+c^2}{c^2-a^2}}+1}\right) - \frac{2}{\sqrt{\xi+c^2}}\right] \quad , \quad c > (a,b)$$

1. **For the spheroid:**

$$I_c = \int_{\xi}^{\infty} \frac{du}{(c^2+u)\sqrt{(a^2+u)(b^2+u)(c^2+u)}}$$

Note that: for the spheroid $a = b$

$$\therefore\ I_c = \int_{\xi}^{\infty} \frac{du}{(c^2+u)\sqrt{(a^2+u)^2(c^2+u)}} = \int_{\xi}^{\infty} \frac{du}{(u+c^2)^{\frac{3}{2}}|u+a^2|}$$

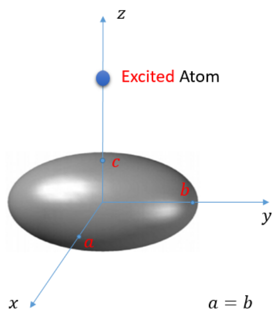

Figure S2. Atom located on z-axis at point z and having dipole momentum oriented along z-axis.

Because of a and b always are $+ve$.

$$\therefore I_c = \int_{\xi}^{\infty} \frac{du}{(c^2+u)\sqrt{(a^2+u)^2(c^2+u)}} = \int_{\xi}^{\infty} \frac{du}{(u+c^2)^{\frac{3}{2}}(u+a^2)}$$

Let: $v = \sqrt{u+c^2} \rightarrow dv = \frac{1}{2\sqrt{u^2+c^2}}\,du \rightarrow du = 2\sqrt{u^2+c^2}\;dv$

$u = v^2 - c^2$ at $u = \xi \rightarrow v = \sqrt{\xi^2+c^2}$

at $u = \infty \rightarrow v = \infty$

$$\therefore I_c = \int_{\sqrt{\xi+c^2}}^{\infty} \frac{2\sqrt{u^2+c^2}\;dv}{(v^2-c^2+c^2)\sqrt{u^2+c^2}(v^2-c^2+a^2)} = \int_{\sqrt{\xi+c^2}}^{\infty} \frac{2\;dv}{v^2(v^2-c^2+a^2)}$$

By partial fraction, we get:

$$\therefore I_c = 2\int_{\sqrt{\xi+c^2}}^{\infty} \left(\frac{1}{(c^2-a^2)(v^2-c^2+a^2)} - \frac{1}{(c^2-a^2)v^2}\right) dv$$

$$= \frac{2}{(c^2-a^2)} \int_{\sqrt{\xi+c^2}}^{\infty} \left(\frac{1}{(v^2-c^2+a^2)} - \frac{1}{v^2}\right) dv$$

- By solving the 1st integral:

$$\int_{\sqrt{\xi+c^2}}^{\infty} \frac{1}{(v^2-c^2+a^2)}\,dv = \int_{\sqrt{\xi+c^2}}^{\infty} \frac{1}{v^2+(a^2-c^2)}\,dv$$

Let: $\omega = \frac{v}{\sqrt{a^2-c^2}} \rightarrow d\omega = \frac{dv}{\sqrt{a^2-c^2}} \rightarrow dv = \sqrt{a^2-c^2}\,d\omega$

$$v = \omega\sqrt{a^2 - c^2} \qquad \text{at} \qquad v = \sqrt{\xi + c^2} \;\rightarrow\; \omega = \sqrt{\frac{\xi + c^2}{a^2 - c^2}}$$

$$\text{at} \qquad v = \infty \qquad \rightarrow \quad \omega = \infty$$

$$\int_{\sqrt{\frac{\xi+c^2}{a^2-c^2}}}^{\infty} \frac{\sqrt{a^2 - c^2}}{(a^2 - c^2)^2\omega^2 + (a^2 - c^2)} d\omega = \frac{1}{\sqrt{a^2 - c^2}} \int_{\sqrt{\frac{\xi+c^2}{a^2-c^2}}}^{\infty} \frac{d\omega}{\omega^2 + 1}$$

$$= \frac{1}{\sqrt{a^2 - c^2}} tan^{-1}(\omega)\Big|_{\sqrt{\frac{\xi+c^2}{a^2-c^2}}}^{\infty} = \frac{1}{\sqrt{a^2 - c^2}}\left[tan^{-1}(\infty) - tan^{-1}\left(\sqrt{\frac{\xi + c^2}{a^2 - c^2}}\right)\right]$$

$$= \frac{1}{\sqrt{a^2 - c^2}}\left[\frac{\pi}{2} - tan^{-1}\left(\sqrt{\frac{\xi + c^2}{a^2 - c^2}}\right)\right]$$

• By solving the 2nd integral:

$$\int_{\sqrt{\xi+c^2}}^{\infty} \frac{1}{v^2} dv = -\frac{1}{v}\Big|_{\sqrt{\xi+c^2}}^{\infty} = -\frac{1}{\infty} + \frac{1}{\sqrt{\xi + c^2}} = \frac{1}{\sqrt{\xi + c^2}}$$

$$\therefore I_c(\xi) = \frac{2}{(c^2 - a^2)}\left[\frac{1}{\sqrt{a^2 - c^2}}\left(\frac{\pi}{2} - tan^{-1}\sqrt{\frac{\xi + c^2}{a^2 - c^2}}\right) - \frac{1}{\sqrt{\xi + c^2}}\right], \; c < (a, b) \qquad \text{for spheroid}$$

Section S2. Radiative decay rate for the ellipsoid and the spheroid using I_c integral

$$\left(\frac{\gamma}{\gamma_0}\right)_z^{rad} = \left|1 + \frac{1}{2}\, abc\,(1 - \varepsilon_{zz})\left(\frac{\varepsilon - 1}{\varepsilon - \varepsilon_{zz}}\right) \times \left(\frac{2}{z\sqrt{(z^2 + a^2 - c^2)(z^2 + b^2 - c^2)}} - I_c(\xi)\right)\right|^2$$

To find the value of ξ if the exited atom is located at z-direction, apply the following equation:

$$\frac{x^2}{a^2+\xi}+\frac{y^2}{b^2+\xi}+\frac{z^2}{c^2+\xi}=1$$

at $x=0$, $y=0$

$$\therefore \frac{z^2}{c^2+\xi}=1 \rightarrow \xi=z^2-c^2$$

Substitute in equation $I_c(\xi)$ we get:

$$\therefore \left(\frac{\gamma}{\gamma_0}\right)_z^{rad}=\left|1+\frac{1}{2}abc\,(1-\varepsilon_{zz})\left(\frac{\varepsilon-1}{\varepsilon-\varepsilon_{zz}}\right)\times\left(\frac{2}{z\sqrt{(z^2+a^2-c^2)(z^2+b^2-c^2)}}-I_c(z^2-c^2)\right)\right|^2$$

For ellipsoid:

$$\therefore I_c(z^2-c^2)=\frac{1}{(c^2-a^2)}\left[\frac{1}{\sqrt{a^2-c^2}}\ln\left(\frac{\sqrt{\frac{z^2}{c^2-a^2}}+1}{\sqrt{\frac{\xi+c^2}{c^2-a^2}}+1}\right)-\frac{2}{z}\right] , \quad c>a \qquad \text{(p.20)}$$

Copyright: © 2021 by the authors. Licensee MDPI, Basel, Switzerland. This article is an open access article distributed under the terms and conditions of the Creative Commons Attribution (CC BY) license (http://creativecommons.org/licenses/by/4.0/).

For spheroid:

$$\therefore i_n(z^2-c^2)=\frac{2}{(c^2-a^2)}\left[\frac{1}{\sqrt{a^2-c^2}}\left(\frac{\pi}{2}-\tan^{-1}\sqrt{\frac{z^2}{a^2-c^2}}\right)-\frac{1}{z}\right] , \quad c<a \qquad \text{(p.21)}$$

Section S3. The general formula the effective medium theory

The general formula of the effective medium theory to calculate the elliptical permittivity and RI is

$$n_{eff}(z)=n_1\,\frac{n_1+\left[L_g\left(1-f(z)\right)+f(z)\right](n_2-n_1)}{n_1+L_g\left(1-f(z)\right)(n_2-n_1)}$$

However for the ellipsoid and the spheroid the factor of L_g is varies in different values, but we can do this approximation at $L_g \to 0$ and $L_g \to 1$:

For ellipsoid: the L_g value approaches to 0 at large aspect ratio, so that the effective permittivity of the ellipsoid could be:

$$n_{eff}(z) = f(z)\, n_2 + (1 - f(z))\, n_1$$

For spheroid: the L_g value approaches to 1 at the small aspect ratio, so that the effective permittivity of the spheroid could be:

$$(n_{eff}(z))^{-1} = f(z)\, n_2^{-1} + (1 - f(z))\, n_1^{-1}$$

Whereas L_g and for the sphere $L_g = 1/3$ this formula is reduced to this equation

$$n_{eff}(z) = n_1 \frac{3\, n_1 + (1 + 2f(z))\,(n_2 - n_1)}{3\, n_1 + (1 - f(z))\,(n_2 - n_1)}$$

For example:

Let $n_1 = 1.5$, $n_2 = 3$ and $f(z) = 0.5$ and by varing the value of L_g form 0 to 1. By applying the general equation of the effective medium theory that mentioned before, we get:

$$n_{eff}(z) = n_1 \frac{n_1 + [L_g(1 - f(z)) + f(z)]\,(n_2 - n_1)}{n_1 + L_g(1 - f(z))\,(n_2 - n_1)} = 1.5\, \frac{1.5 + [L_g(1 - 0.5) + 0.5]\,(3 - 1.5)}{1.5 + L_g(1 - 0.5)\,(3 - 1.5)}$$

$$n_{exact} = \frac{0.75\; L_g + 2.25}{0.5\, L_g + 1}$$

$$n_{approxi} = 0.5\,(3) + (1 - 0.5)\,1.5 = 2.25.$$

the exact solution for large aspect ratio $c/a = 5 \to L_g = 0.0558$ using the formula in the attached paper Equation (9),

the $n_{exact} = 2.216$, so that in order to calculate the error $\Delta\varepsilon\%$:

$$\Delta \varepsilon \% = \left|\frac{n_{exact} - n_{approxi}}{n_{exact}}\right| * 100\%$$

$$= \left|\frac{2.216 - 2.25}{2.216}\right| * 100\% = 1.534\ \%.$$

Which means that the approximation could be acceptable in this range.
